# Evaluation of a new whole room indirect calorimeter specific for measurement of resting metabolic rate

**DOI:** 10.1186/s12986-015-0043-0

**Published:** 2015-11-19

**Authors:** Russell Rising, Kathryn Whyte, Jeanine Albu, Xavier Pi-Sunyer

**Affiliations:** New York Obesity Research Center, Department of Medicine, Columbia University, 1150 St. Nicholas Ave, 1st Floor, Suite 121, New York, NY 10032 USA; 46 Meadowbrook Drive, Apt 121, Slingerlands, NY 12159 USA; Icahn School of Medicine at Mount Sinai, 1111 Amsterdam Avenue, New York, NY 10025 USA

**Keywords:** Resting metabolic rate, energy expenditure, metabolic cart, whole room indirect calorimeter, ventilated hood

## Abstract

**Background:**

The most common methods for obtaining human resting metabolic rate (RMR) use either a ventilated hood connected to a metabolic cart (VH_MC) or calculation by many prediction equations utilizing the person’s height and weight. These methods may be inherently inaccurate. The objective of this study is to compare the accuracy for the measurement of RMR by three methods: a new whole room indirect calorimeter specific for this purpose (RMR_WRIC), VH_MC and calculation by the Mifflin equation (ME).

First, the VH_MC (Vmax Encore 2900, Carefusion Inc, San Diego, CA) and RMR_WRIC (Promethion GA-6/FG-1, Sable Systems Intl, Las Vegas, NV) were subjected to 10, one-hour ethanol (99.8 % purity) and propane (99.5 % purity) combustion tests, respectively, for simulated metabolic measurements. Thereafter, 40 healthy adults (22 M/18 F, 78.0 ± 24.5 kg, BMI = 25.6 ± 4.8, age 36.6 ± 13.4 years) had one-hour RMR (kcal), ventilation (liters) rates of oxygen (VO_2_), carbon dioxide (VCO_2_) and RQ (VCO_2_/VO_2_) measured after a 12-h fast with both the VH_ MC and the RMR_WRIC in a randomized fashion. The resting state was documented by heart rate. The RMR was also calculated using the ME, which was compared to both the RMR_WRIC and the VH_MC. All simulated and human metabolic data were extrapolated to 24-h and analyzed (SPSS, Ver. 22).

**Results:**

Comparing stoichiometry to actual combustion, the VH_MC underestimated simulated RMR (*p* < 0.05), VO_2_ (*p* < 0.05), VCO_2_ (*p* < 0.05) and the RQ. Similarly the RMR_WRIC underestimated simulated RMR (*p* < 0.05) and VO_2_ while overestimating VCO_2_ and the RQ. There was much greater variability in the simulated metabolic data between combustion and the VH_MC as compared to that of the RMR_WRIC. With regards to the volunteers, the RMR, RQ, VO_2_ and VCO_2_ determined by the VH_MC tended to be lower in comparison to these measurements determined by the RMR_WRIC. Finally, RMR calculated utilizing the ME was significantly (*p* < 0.05) less than the RMR_WRIC but similar to that obtained by the VH_MC.

**Conclusion:**

The RMR_WRIC was more accurate and precise than either the VH_MC or ME, which has implications for determining energy requirements for individuals participating in weight loss or nutrition rehabilitation programs.

## Background

Inaccurate short duration RMR measurements commonly done in many research and clinical health care institutions can lead to large errors in extrapolated 24-h sedentary energy requirements. This is a report of a new indirect calorimetric method that is both accurate and comfortable for human subjects thereby eliminating many of the problems that are associated with short-duration metabolic measurement techniques.

Indirect calorimetry has made significant contributions to the knowledge of energy needs of humans. For example, the effects of aging [1], changes in body composition [2], ethnic group [3] and gender [4] have been found to affect energy requirements. The most common clinical application of indirect calorimetry is the measurement of energy expenditure over a short period of time, usually between 30 to 45 min, utilizing a VH_MC. Extrapolated 24-h RMR is then calculated from these short duration measurements. Since RMR accounts for approximately 70 % of total daily EE [5], this has been the main calorimetric technique utilized in both health care and research settings [6].

The first practical clinical measurements of RMR were obtained in 1918 by the respiratory-valve and spirometer (Tissot) technique [7]. This device required subjects to wear a tight fitting face mask during measurements. The early 1980’s saw several systems developed in an attempt to obtain more accurate RMR measurements. For example, the Beckman metabolic cart still required subjects to wear a face mask during measurements [8]. Other systems were custom fabricated from assembled electronic components [9] but still necessitated that subjects be placed under a ventilated hood during measurements. The Deltatrac metabolic monitors were the first single module units produced but still required ventilated hoods [10]. The Deltatrac Metabolic Monitor became the gold standard by which other new techniques for RMR measurement were compared [11–13]. However the Deltatrac metabolic monitors were discontinued in 2007 and were replaced by the Sensormedic VMax 2900 metabolic cart [14] as the new gold standard for RMR measurements.

A major disadvantage of all the past and present metabolic carts is the necessity for the use of ventilated hoods, face masks or nose clips that may cause discomfort or anxiety in subjects [15]. This can be compared to the claustrophobic nature of an MRI scanner which has been found to induce a cortisol response in patients during measurements [16]. Subject anxiety or discomfort during measurement may be reflected in an elevated RMR.

To eliminate these problems, a new WRIC was designed and built specifically for the measurement of RMR in humans. The present report evaluates the accuracy of RMR measurements with the new RMR_WRIC and compares it to the VH_MC and to that calculated with the ME [17] using the gold standards ethanol for the VH_MC and propane combustion [5] for the RMR_WRIC. This study demonstrates that RMR measured by the RMR_WRIC is more accurate than the VH_MC or that calculated by the ME [17]. The accuracy of the RMR_WRIC, VH_MC and the ME is presented in regards to both the gold standards and in human subjects.

## Methods

### Description of the new RMR_WRIC

**Fig. 1:**
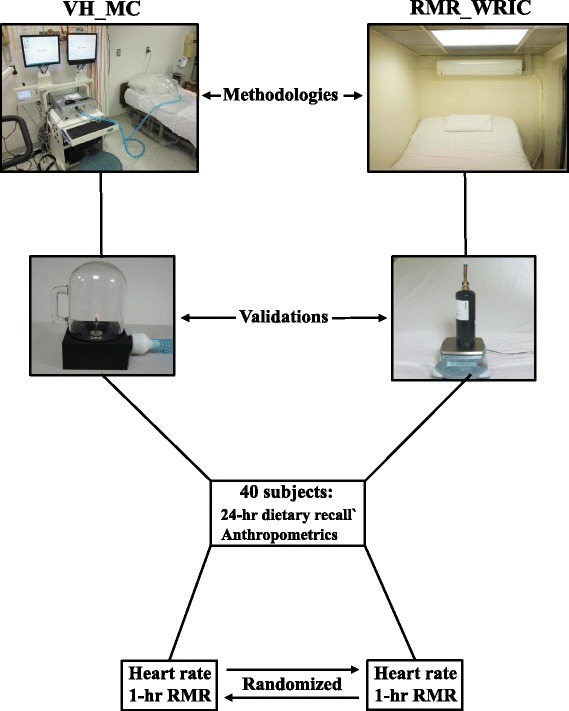
Schematic representation of the experimental procedures utilized to validate the new whole room indirect calorimeter, specific for measuring resting metabolic rate (RMR_WRIC), against a ventilated hood connected to a metabolic cart (VH_MC). Accuracy and precision of each methodology was first obtained through ethanol (VH_MC) and propane (RMR_WRIC) combustion tests and comparison to their related stoichiometries. Finally, both methodologies were utilized for measurement of resting metabolic rate in 40 healthy adult subjects

The RMR_WRIC at the New York Obesity Nutrition Research Center in Manhattan, NY is a 4,500 liter climate controlled room equipped with a standard size hospital bed (91 × 203 × 20 cm) and a small stereo for music entertainment. A large window and an intercom system are available so family members and investigators can observe and talk to the subject during RMR measurements. This new RMR_WRIC eliminates the need for any kind of head apparatus. The respiratory exchange is measured by the Promethion (model GA-06/ FG-01) integrated whole room indirect calorimeter system (Sable Systems International, Las Vegas NV). This is accomplished by flowing a fixed 100 L/min of fresh air through the RMR_WRIC chamber and obtaining a sample on the exhaust side of the system for measurement of O_2_ and CO_2_ concentrations (%). This research grade instrumentation is accurate to 0.001 % for mass air flow (liters), O_2_ and CO_2_ concentrations. Moreover, WVP of the sample gas stream is measured directly to 0.001 Kpa and utilized to continuously correct the VO_2_ and VCO_2_, along with mass air flow (L). This eliminates the need for any type of desiccants to dry the sample gas stream during metabolic measurements. The lack of desiccants eliminates any potential errors due to incomplete removal of moisture prior to analysis of sample gases and mass air flow [18]. Moreover, an instantaneous z-transformation mathematical model is applied to the continuous O_2_ and CO_2_ gas concentrations in order to account for the corrected room volume of 4,500 L in their response time to metabolic changes of the subject [19]. Energy expenditure (kcal), VO_2_ and VCO_2_, along with the RQ (VCO_2_/VO_2_) are then calculated (20) every minute during the RMR measurement. A schematic of the entire experimental protocol is shown in Fig. 1.

### Validations

Ten one-hour ethanol (99.8 % purity; Fisher Scientific, Pittsburgh, PA) combustion tests were performed utilizing a burn kit (#769137, Carefusion Inc. San Diego, CA) to determine the accuracy and precision of the VH_MC (Vmax Encore, Carefusion Inc. San Diego, CA). This was accomplished by comparing the stoichiometry of ethanol for EE (7.12 kcal/g), VO_2_, VCO_2_ and the RQ of 0.67 to that obtained through actual combustion. Similarly, 10 one-hour propane (99.5 % purity; Air Liquide, Houston, TX) combustion tests were performed to determine the accuracy and precision of the RMR_WRIC. This was accomplished by comparing the stoichiometry of propane for EE (11.92 kcal/g), VO_2_, VCO_2_ and the RQ of 0.60 to that obtained through actual combustion. Ethanol [20] and propane [5] combustion are accepted gold standards for validating VH_MC’s and WRIC’s, respectively.

To simulate human subject protocols for RMR measurements, each one-hour ethanol or propane combustion test was comprised of both a 15 and 45-min acclimation and measurement periods, respectively. Mean EE across the 45-min measurement period was then multiplied by 1440 (minutes in 24-h) to obtain extrapolated 24-h simulated RMR (kcal/d) for both methods. The burn rate (g/min) for both ethanol and propane were determined by obtaining the weight prior to and after completion of each combustion test using a calibrated analytical balance (Adventurer Pro Model AV812, Ohaus Corporation, Pine Brook, NJ). The accuracy of the analytical balance was confirmed prior to the initiation of each ethanol or propane combustion test by placing calibration weights of the expected weight ranges on the balance. Prior to the commencement of the combustion tests, the instrumentation related to both the VH_MC and the RMR_WRIC were calibrated according to each respective manufacturer’s instructions. From the burn rates, the EE was calculated every minute utilizing the Weir equation [21], along with VO_2_, VCO_2_ and RQ by the Vmax program manager (Ver. 21-1A) for the VH_MC and Expedata (Ver.1.4.15) for the RMR_WRIC. The results from each combustion test were then compared to that calculated from the respective stoichiometry.

### Human volunteer subjects

Forty healthy non-smoking adult subjects (22 M/18 F, 78.0 ± 24.5 kg, BMI = 25.6 ± 4.8, age 36.6 ± 13.4 years) were recruited by word of mouth to participate in this study. Medical histories were obtained by physicians familiar with the study. Exclusion criteria included age < 18 years old, recent or prescribed medications affecting metabolism, pregnancy or lactation as verified by an on-site HCG urine test, unstable weight maintenance within the last three months and reported or diagnosed claustrophobia.

After informed consent was obtained, subjects were instructed to fast for 12-h and refrain from caffeine, strenuous exercise and alcohol for one day prior to their scheduled morning appointment. Anthropometrics were obtained upon arrival to the laboratory. Prior to the first RMR measurement (VH_MC or RMR_WRIC), heart rate (beats/min) was measured by wrist palpation after a one-hour supine rest to determine each subject’s baseline (resting) state. The subjects were randomized as to which of the two methods (VH_MC or RMR_WRIC) was used first. A registered dietitian recorded 24-h food recalls for the period prior to the appointment time to ensure a fasting state. Similar to the ethanol or propane combustion tests, each metabolic measurement was comprised of acclimation and measurement periods and EE was extrapolated to 24-h RMR.

The VH_MC and RMR_WRIC are located in two different laboratories on the same floor within 20 m of each other. After the first RMR measurement was completed, subjects were escorted to the next laboratory, where they rested for at least 30 min. Thereafter, heart rate was taken again in triplicate and averaged to assure a baseline state similar to the initial one. The second RMR measurement was not started until the interim average heart rate was within five beats per minute of that obtained initially. Prior to the start of each RMR measurement, respective instrumentations were calibrated as described previously. During RMR measurements subjects were asked to remain awake, relax and minimize any movements while lying in a comfortable supine position under the ventilated hood of the metabolic cart or on a standard hospital bed in the RMR_WRIC. Subjects were monitored during each of the entire one-hour RMR measurements to ensure compliance to study procedures. This is especially necessary for proper functioning of the VH_MC as this methodology utilizes a manually adjusted flow rate. The flow rate of the VH_MC was constantly monitored and adjusted to maintain exhaled CO_2_ levels below 1.0%. The RMR_WRIC uses a pre-set continuous flow rate of 100 L/min and no manual adjustments were necessary as the CO_2_ levels within the chamber never exceeded 0.50 %. Collected raw data for the subjects were analyzed by each respective method’s instrumentation (VH_MC and RMR_WRIC) software as described above for the ethanol and propane combustion tests. Finally, the ME [17] [24-h RMR, kcal = ((9.99 × body weight, kg) + (6.25 × height, cm) - (4.92 × age, years) + (166 * sex, males = 1, females = 0) -161)] was utilized to calculate predicted 24-h RMR for each subject for comparison to the RMR_WRIC and VH_MC.

Study procedures were approved by the Institutional Review Boards of both St. Luke’s-Roosevelt Hospital and Columbia University, and were conducted in accordance with the Declaration of Helsinki of 1975 as revised in 1983. All participants provided written informed consent before enrollment.

### Statistics

Power calculations [22] were performed utilizing data from a previous study [23] to determine the number of subjects required for detection of any statistical differences in RMR between the two indirect calorimetry methods. Statistical analysis was performed using SPSS (Version 22, Chicago, IL). Independent t-tests were used to determine any effects of which method was used first for RMR measurements. Paired t-tests (*p* < 0.05) were utilized to determine any differences in all metabolic parameters between ethanol or propane stoichiometry and that obtained from actual combustion for both the VH_MC and the RMR_WRIC. A similar analysis was performed to determine any differences between both methodologies for all metabolic parameters measured in the human subjects. Moreover, this same analysis was performed to determine any differences between RMR measured by both the RMR_WRIC and VH_MC to that calculated by the ME [17]. Pearson Correlations were utilized to determine the relationships between all metabolic parameters obtained from both the VH_MC and the RMR_WRIC. Fresh air flow rates were not compared using t-tests due to the differences in the principles behind their design and utilization for both the VH_MC and RMR_WRIC.

The Bland-Altman [24] limit analysis was applied first to the ethanol and propane data to determine agreement between related stoichiometry and actual combustion in regards to simulated RMR, RQ, VO_2_ and VCO_2_ for both the VH_MC and RMR_WRIC. Furthermore, a similar analysis was applied to the subject data to determine the magnitude of agreement for these same metabolic parameters between the two methods. Moreover, proportional bias (*p* < 0.05) was determined by regressing mean differences for combustion (ethanol or propane) for each the VH_MC and the RMR_WRIC for RMR, VO_2_ and VCO_2_ on mean values obtained by both methods. A similar proportional bias was also performed for the human subject data utilizing mean differences in the metabolic parameters obtained with each methodology. Finally, the Bland-Altman limit analysis [24] was applied to the RMR data obtained by the RMR_WRIC and that calculated with the ME [17] to determine agreement between an actual measurement method and a prediction equation.

## Results

### Validations

**Fig. 2:**
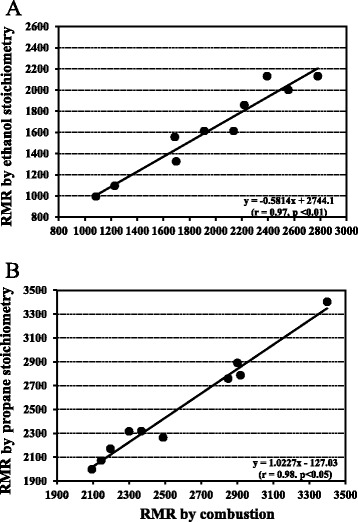
Relationships between 24-h resting metabolic rate (RMR; kcal/d) calculated from ethanol and propane stoichiometries and that obtained from combustion for the ventilated hood connected to the metabolic cart (VH_MC; plot **a**) and for the whole room indirect calorimeter specific for measuring resting metabolic rate (RMR_WRIC; plot **b**)

All descriptive statistics for simulated RMR (kcal/d), RQ, (VCO_2_/VO_2_), VO_2_ (L/min) and VCO_2_ (L/min) calculated from ethanol and propane stoichiometry and that obtained by combustion for each method are shown in Table 1. The VH_MC tended to underestimate while the RMR_WRIC produced mean RQs’ slightly, but not significantly, over the stoichiometric values for ethanol and propane (Table 1). Simulated RMR’s, calculated from both ethanol and propane stoichiometry, were both correlated (*p* < 0.05) to that obtained from actual combustion of the VH_MC (Fig. 2a) and RMR_WRIC (Fig. 2b). The related comparisons in terms of the Bland-Altman limit analysis are presented in Table 2. The magnitude of the differences were greater (*p* <0.05) for simulated RMR, VO_2_ and VCO_2_ between ethanol stoichiometry and that obtained from actual combustion for the VH_MC as reflected by the limits of agreement shown in Table 2. This was also reflected in a much greater bias and 95 % confidence intervals (Table 2). The magnitude of the differences between propane stoichiometry and combustion for simulated RMR, VO_2_ and VCO_2_ obtained from the RMR_WRIC were of smaller magnitude as reflected by the much tighter limits of agreement as shown in Table 2. The agreement for the RQ between ethanol and propane stoichiometry and that obtained from combustion for each method were similar. Moreover, no proportional bias existed for RQ with either method, suggesting that larger average values do not lead to greater disagreements between stoichiometry and combustion (Table 2). However, there was significant proportional bias for simulated RMR, VO_2_ and VCO_2_ for the VH_MC while none existed for these values for the RMR_WRIC. This suggests that greater disagreements will occur with larger average values when using the VH-MC for measurements of RMR, VO_2_ and VCO_2_.

**Table 1:** Descriptive statistics for all metabolic variables measured with each technique

	N	Mean	Min	Max	± SD	SE	Mean	Min^2^	Max^3^	± SD	SE
VH_MC^1^		Calculated from ethanol stoichiometry					Derived from ethanol combustion				
Burn rate (g/min)	10	0.193	0.106	0.272	0.054	0.017	-----	-----	-----	-----	-----
RMR^4^ (kcal/d)	10	1969.1	1083.7	2780.9	554.2	175.3	1631.5	993.3	2131.2	406.0	128.4
RQ^5^ (VO_2_/VCO_2_)	10	0.67	0.67	0.67	0.001	0.002	0.66	0.65	0.67	0.007	0.002
VO_2_^6^ (L/day)	10	405.2	223.0	572.2	114.0	36.1	360.2	218.9	472.3	89.6	28.3
VCO_2_^7^ (L/day)	10	270.1	148.7	381.5	76.0	24.0	238.0	141.1	312.5	60.3	19.0
RMR_WRIC^8^		Calculated from propane stoichiometry					Derived from propane combustion				
Burn rate (g/min)	10	0.150	0.122	0.198	0.025	0.008	-----	-----	-----	-----	-----
RMR (kcal/d)	10	2566.1	2094.1	3398.7	429.6	135.8	2497.5	1997.0	3402.8	445.2	140.8
RQ (VO_2_/VCO_2_)	10	0.60	0.60	0.60	0.00	0.00	0.61	0.59	0.62	0.01	0.00
VO_2_ (L/day)	10	548.0	447.2	725.8	91.2	29.0	540.5	433.2	735.2	95.6	30.2
VCO_2_ (L/day)	10	328.8	268.3	435.5	55.0	17.4	332.2	261.9	456.8	62.2	19.7
Subjects		Measured by VH_MC					Measured by RMR_WRIC				
RMR (kcal/d)	40	1701.7	964.8	2390.4	322.5	51.0	1896.6	1123.2	2865.6	392.4	62.0
RQ (VO_2_/VCO_2_)	40	0.81	0.73	0.93	0.04	0.01	0.83	0.75	0.97	0.05	0.01
VO_2_ (l/day)	40	352.4	199.1	510.0	68.6	10.9	396.6	230.0	595.9	81.2	12.8
VCO_2_ (l/day)	40	284.4	162.0	373.7	51.6	8.2	319.7	195.7	470.0	65.1	10.3

^1^ *VH_MC* Ventilated hood connected to a metabolic cart

^2^ *Min* Minimum value

^3^ *Max* Maximum value

^4^ *RMR* Resting metabolic rate

^5^ *RQ* Respiratory quotient

^6^ *VO*_*2*_ Ventilation rate of oxygen

^7^ *VCO*_*2*_ Ventilation rate of carbon dioxide

^8^ *RMR_WRIC* Resting metabolic rate whole room indirect calorimeter

**Table 2:** Bland-Altman limit analysis for comparison between the VH_MC and RMR_WRIC for ethanol and propane combustion along with subject metabolic testing

Comparison	Parameter	Bias	95 % CI	Limits of agreement	Proportional bias (*p* < 0.05)
Ethanol Vs. VH_MC^1^	RMR^3^ (kcal/d)	337.6 ± 192.6	2.7 - 672.5	−47.6 - 722.8	<0.01
	RQ^4^ (VO_2_/VCO_2_)	0.01 ± 0.01	0.00 - 0.02	0.00 - 0.020	NS
	VO_2_^5^ (l/day)	45.0 ± 35.6	0.2 - 89.8	−26.2 - 116.2	<0.01
	VCO_2_^6^ (l/day)	32.1 ± 23.9	0.2 - 64.0	−15.7 - 79.9	<0.01
Propane vs.^2^ RMR_WRIC	RMR (kcal/d)	68.7 ± 72.8	−0.3 - 137.7	−76.9 - 214.3	NS
	RQ (VO_2_/VCO_2_)	−0.01 ± 0.01	0.00 - 0.02	−0.030 - 0.010	NS
	VO_2_ (l/day)	7.49 ± 15.90	0.00 - 15.00	−24.31 - 39.29	NS
	VCO_2_ (l/day)	−3.38 ± 12.00	−6.80 - 0.00	−27.38 - 20.62	NS
VH_MC vs. RMR_WRIC^8^	RMR (kcal/d)	194.9 ± 170.2	1.20 - 388.6	−145.5 - 535.3	<0.01
	RQ (VO_2_/VCO_2_)	0.02 ± 0.05	0.00 - 0.04	−0.08 - 0.12	NS
	VO_2_ (l/day)	−44.20 ± 41.90	−88.4 - 0.00	−128.00 - 39.6	<0.05
	VCO_2_ (l/day)	−35.30 ± 30.64	−70.3 - -0.30	−96.5 - 25.90	<0.01

^1^ *VH_MC* Ventilated hood connected to a metabolic cart

^2^ *vs*. Verses

^3^ *RMR* Resting metabolic rate

^4^ *RQ* Respiratory quotient

^5^ *VO*_*2*_ Ventilation rate of oxygen

^6^ *VCO*_*2*_ Ventilation rate of carbon dioxide

^7^ *RMR_WRIC* Resting metabolic rate whole room indirect calorimeter

### Human subjects

**Fig. 3:**
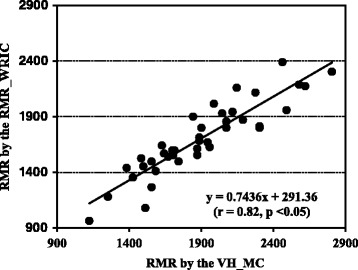
Relationship between the 24-h resting metabolic rate (RMR; kcal/d) measured in 40 healthy adult non-smoking human subjects by both the ventilated hood connected to the metabolic cart (VH_MC) and the whole room indirect calorimeter specific for measuring resting metabolic rate (RMR_WRIC)

**Fig. 4:**
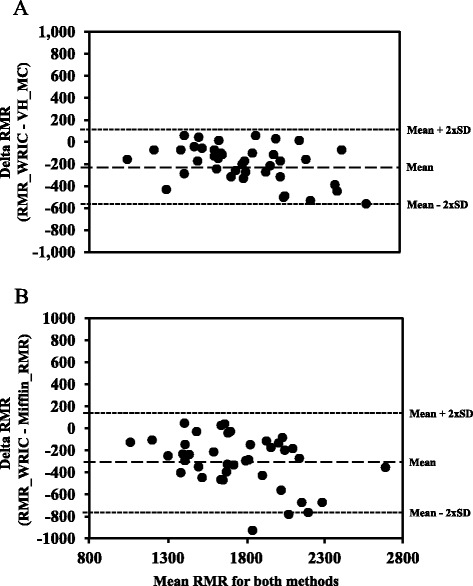
Bland-Altman limits of agreement analysis for RMR (RMR; kcal/d) between the ventilated hood connected to the metabolic cart (VH_MC) and the whole room indirect calorimeter specific for measuring resting metabolic rate (RMR_WRIC; plot **a**) and the RMR_WRIC and RMR calculated with the Mifflin equation (plot **b**) for the 40 healthy adult non-smoking human subjects

All subjects were at a resting state prior to each metabolic measurement as reflected by lack of significant differences in their pre (59.1 ± 9.2) and post (58.6 ± 10.1) metabolic test heart rates (beats/min). Moreover, the sequence to which method was performed first had no effect upon any of the metabolic measurements (p = 0.36). With regard to the measurements obtained by both methods, no correlation was found for the RQ. However, RMR measured by both methodologies (Fig. 3) were correlated (*p* < 0.05). In comparison to the RMR_WRIC, the VH_MC underestimated RMR, RQ, VO_2_ and VCO_2_ by 9.7 ± 7.8, 2.6 ± 5.7, 10.5 ± 9.7 and 10.4 ± 8.0 %, respectively. This is further reflected in terms of the Bland-Altman limit analysis as shown in Table 2. Furthermore, the Bland-Altman limits of agreement plot clearly shows how RMR is underestimated by the VH_MC in comparison to the RMR_WRIC (Fig. 4a). Moreover, there was significant proportional bias for all metabolic measurements except the RQ, suggesting that greater values will lead to larger disagreements between the two methods (Table 2).

Finally, in comparison to RMR measured in the human subjects by both the RMR_WRIC (1896.6 ± 392.4 kcal/day) and the VH_MC (1701 ± 322.5 kcal/day), the Mifflin equation underestimated their RMR (1601.4 ± 306.3 kcal/day) by 14.7 ± 10.1 (*p* < 0.05) and 5.30 ± 10.13 %, respectively. This is further reflected in the Bland-Altman limit analysis plot (Fig. 4b) for the RMR_WRIC vs the ME showing wide limits of agreement.

## Discussion

When compared to ethanol stoichiometry, there was greater variation in terms of RMR, VO_2_ and VCO_2_, as well as the RQ with the VH_MC versus that compared with propane stoichiometry for the RMR_WRIC. This was verified by the Bland-Altman limit analysis which showed much greater limits of agreement as well as much wider confidence intervals for the VH_MC. All of the metabolic measurements during combustion validations with the RMR_WRIC were of greater accuracy and precision than that obtained for the VH_MC when compared to the respective stoichiometries. With regard to the comparison between the two methods for the human subjects, all metabolic measurements, except the RQ, were greater for the RMR_WRIC. Even though there was a relationship between the two methods in terms of RMR, the values obtained in the human subjects by the RMR_WRIC tended to be 10 % greater than that from the VH_MC. Similar results were found for the RQ with a 5 % over estimate.

As noted in the results for the human subjects, significant differences were found between the two methods for RMR measurements with lower values obtained by the VH_MC. This is in contrast to the expected where RMR measured by the VH_MC would be greater due to the possibility of subject anxiety while being placed under the ventilated hood [15, 16]. The elimination of the ventilated hood, along with a more comfortable environment of the RMR_WRIC chamber, suggests that RMR would be lower with this technique. However, the opposite was found. If ethanol and propane combustion and related stoichiometry’s are utilized as the standard, the RMR_WRIC showed greater accuracy and precision for metabolic measurements than the VH_MC.

The unexpected lower values for RMR with the VH_MC might be partly explained by the technological and procedural differences between the two methods. For example, the fresh air flow rates utilized for the RMR-WRIC is fixed at 100 L/min throughout the one-hour RMR measurement. This is in contrast to the VH_MC where the fresh air flow rate is constantly adjusted to maintain carbon dioxide levels below one percent while the subject is under the ventilated hood. Adjustments of greater magnitude are necessary for extremely large or obese subjects due to the small volume of the ventilated hood thus possibly compounding the errors. These constant adjustments to the fresh air flow rate might produce consistent errors by the VH_MC systems electronics. Therefore, more adjustments to the fresh air flow may translate to a greater magnitude of errors in RMR. However, this problem is eliminated with the RMR_WRIC. This is similar to the first Deltatrac Metabolic Monitors which also used a single fresh air flow setting of 40 L/min [25] during RMR measurements.

Not accounting for all of the sample gas moisture content prior to the measurement of O_2_ and CO_2_ concentrations can lead to underestimates in calculated RMR. This might partly explain the underestimated RMR with the VH_MC. The early Deltatrac Metabolic Monitors, as well as VH_MC’s used today, rely on Naphion tubing to partly remove moisture from the sample gas stream prior to analysis. Naphion tubing only equilibrates the sample gas moisture content to that of the ambient air within the lab and an additional calculation for sample gas moisture content is utilized by the VH_MC’s software to further correct for its effects. Poor maintenance of the Naphion tubing can contribute to inadequate equilibration thus contributing to additional errors in metabolic calculations.

Both methods utilize the Weir equation [21] for calculation of energy expenditure. Furthermore, any minor errors in the minute-by-minute calculation of energy expenditure are further amplified upon extrapolation to 24-h. The ventilation rate of oxygen has a threefold greater influence than carbon dioxide on the outcome for RMR with regard to the Weir equation [21]. Therefore, any moisture not accounted for prior to analysis by the O_2_ sensor will lead to large underestimates of the ventilation rates of O_2_ thereby lowering RMR. Similar errors will occur for the ventilation rates of CO_2_ but are of a lesser magnitude due to less of an effect of sample gas moisture on the CO_2_ sensor. Lack of accurate accounting for moisture in the sample gas stream possibly contributed to counteracting the suspected increase in RMR with the VH_MC. This, combined with the potential for errors in the constantly changing fresh air flow rate through the ventilated hood, probably contributed to the overall underestimates of RMR in comparison to the RMR_WRIC. Moreover, this is further verified by the greater variability in all of the metabolic parameters measured by the VH_MC compared against ethanol versus that found for the RMR_WRIC against propane stoichiometry.

All of these potential problems with the VH_MC are eliminated when RMR is measured with the RMR_WRIC utilizing the Promethion integrated instrumentation. This instrumentation does not require the removal of the moisture content of the sample gas stream since it is continuously measured and corrected for at the point of detection. Finally, the single rate fresh air flow setting eliminates any errors by the systems electronics that might occur. These technological improvements, along with improved human subject comfort of the RMR-WRIC chamber, may combine to reduce overall errors in the measurement of RMR.

## Conclusions

The RMR_WRIC is more accurate and precise in the determination of all of the metabolic parameters while providing greater comfort for the human subjects.

## Abbreviations

VH_MC: Ventilated hood connected to a metabolic cart
RMR_WRIC: Resting metabolic rate whole room indirect calorimeter
RMR: Resting metabolic rate
WRIC: Whole room indirect calorimeter
O_2_: Oxygen
CO_2_: Carbon dioxide
L/min: Liters per minute
EE: Energy expenditure
RQ: Respiratory quotient
VO_2_: Ventilation rate of oxygen
VCO_2_: Ventilation rate of carbon dioxide
WVP: Water vapor pressure
ME: Mifflin equation
Kpa: Kilopascals
